# Potential Simultaneous Inhibitors of Angiotensin-Converting Enzyme 2 and Transmembrane Protease, Serine 2

**DOI:** 10.3389/fphar.2020.584158

**Published:** 2020-12-17

**Authors:** Ching-Yuan Wu, Yu-Shih Lin, Yao-Hsu Yang, Li-Hsin Shu, Yu-Ching Cheng, Hung Te Liu

**Affiliations:** ^1^ Department of Traditional Chinese Medicine, Chang Gung Memorial Hospital, Chiayi, Taiwan; ^2^ School of Chinese Medicine, College of Medicine, Chang Gung University, Chiayi, Taiwan; ^3^ Department of Pharmacy, Chiayi Chang Gung Memorial Hospital, Chiayi, Taiwan; ^4^ Health Information and Epidemiology Laboratory of Chang Gung Memorial Hospital, Chiayi, Taiwan

**Keywords:** SARS-CoV-2, ACE2, TMPRSS, theaflavin 3-gallate, theaflavin, (+)-catechin 4

## Abstract

Outbreak of coronavirus disease 2019 occurred in Wuhan and has rapidly spread to almost all parts of world. GB-1, the herbal formula from Tian Shang Sheng Mu of Chiayi Puzi Peitian Temple, is used for the prophylaxis of SARS-CoV-2 in Taiwan. In this study, we investigated that the effect of GB-1 and the index compounds of GB-1 on the ACE2 and TMPRSS2 expression through *in vitro* and *in vivo* study. In our result, GB-1 can inhibit ACE2 and TMPRSS2 protein expression in HepG2 cells, 293T cells, and Caco-2 cells without cytotoxicity. For the mouse model, GB-1 treatment could decrease ACE2 and TMPRSS2 expression levels of the lung and kidney tissue without adverse effects, including nephrotoxicity and hepatotoxicity. In the compositions of GB-1, 0.5–1 mg/ml of *Glycyrrhiza uralensis* Fisch. ex DC*.* extract could not inhibit *ACE2* mRNA and protein expression in HepG2 cells. In addition, theaflavin-3-gallate could inhibit protein expression of ACE2 and TMPRSS2 without significant cytotoxicity. Our results suggest that GB-1 and theaflavin-3-gallate could act as potential candidates for prophylaxis or treatment of SARS-CoV-2 infection through inhibiting protein expression of ACE2 and TMPRSS2 for the further study.

## Introduction

The outbreak of severe acute respiratory syndrome coronavirus 2 (SARS-CoV-2)–induced coronavirus disease 2019 (COVID-19) was recorded in December 2019. About ten million patients across the globe were infected within several months. Angiotensin-converting enzyme 2 (ACE2) is a membrane-associated enzyme for catalyzing the cleavage of angiotensin I to angiotensin (1–9) (Donoghue et al., 2000). Its peptidase domain can directly bind to the receptor-binding domain of the spike protein on the surface of the SARS-CoV-2 viral envelope, thus promoting viral entry into host cells (Hoffmann et al., 2020b; Walls et al., 2020; Yan et al., 2020). After binding to the ACE2 receptor, transmembrane protease, serine 2 (TMPRSS2), and furin of host cells can cleave and activate the SARS-CoV-2 spike protein (Hoffmann et al., 2020a; Hoffmann et al., 2020b).

ACE2 expression and distribution in different parts of the human body might indicate infection routes of SARS-CoV-2. Studies have identified high ACE2 expression in the lung, kidney, intestines, heart, and brain (Baig et al., 2020; Zou et al., 2020). These organs should be regarded as high-risk sites for potential SARS-CoV-2 infection. A recent study described high ACE2 expression in the oral cavity, indicating that the oral route is particularly relevant to SARS-CoV-2 infection (Xu et al., 2020). Therefore, ACE2 and TMPRSS2 are potential antiviral intervention targets for the prevention or SARS-CoV-2 infection or related treatment (Hoffmann et al., 2020b; Danser et al., 2020; Gurwitz, 2020; Morse et al., 2020).

Some herbal formulas are used for the prophylaxis and treatment of SARS-CoV-2 (Li et al., 2020; Ren et al., 2020; Runfeng et al., 2020; Zhang et al., 2020). However, the actual inhibitory mechanisms preventing SARS-CoV-2 entry into the host cells and the efficiency of these formulas remain unclear. In this study, we investigated the effect of GB-1, a formula from Tian Shang Sheng Mu of Chiayi Puzi Peitian Temple, and their index compounds, theaflavin-3-gallate, theaflavin, and (+)-catechin, on the protein and mRNA expression of ACE2 and TMPRSS2.

## Materials and Methods

### Cell Culture and Treatment

The design of the GB-1 formula was obtained from Tian Shang Sheng Mu of Chiayi Puzi Peitian Temple: dry roots of *Glycyrrhiza uralensis* Fisch. ex DC. (25 g; Chang Gung Memorial Hospital, Taiwan) and *Camellia sinensis* var. assamica (black tea, 5 g; Chang Gung Memorial Hospital, Taiwan). This GB-1 or 10 g of the dry root of *G. uralensis* Fisch. ex DC. alone was soaked in 2,000 ml of boiling hot water for 25 min in thermal flasks. The samples were then filtered using a filter paper to remove particulate matter. The obtained water extracts were then evaporated under reduced pressure to obtain viscous masses of 4 g for GB-1 and 3 g for *G. uralensis* Fisch. ex DC. alone. These samples were stored at −80 °C until further experimentation. For all experiments, the final concentrations of the test compounds were prepared by diluting the viscous masses with water. We purchased theaflavin-3-gallate and theaflavin from ChromaDex (Irvine, CA, United States) and (+)-catechin from Sigma-Aldrich.

HepG2 cells, Caco-2 cells, and 293T cells were obtained from the Bioresource Collection and Research Center, Taiwan (passage number of both cell lines = 10–15). HepG2 cells and 293T cells were cultured in Dulbecco’s modified Eagle medium supplemented with 10% fetal bovine serum (FBS) at 37 °C and 5% CO_2_. Caco-2 cells were cultured in Eagle’s minimal essential medium supplemented with 10% fetal bovine serum (FBS) at 37 °C and 5% CO_2_. Before treatment, the cells were cultured to 60–70% confluence, after which the medium was replaced with the same fresh medium in water or methanol at the indicated concentrations. The cells treated with water or vehicle alone were used as untreated controls, whereas the parental cells without any treatment were used as blank controls.

### XTT Assay

The indicated cells were plated at a density of 1 × 10^3^ cells/well in 96-well plates with medium containing 10% FBS. After the cells attached to culture dishes, the medium was replaced with fresh medium containing 10% FBS. The cells were then treated with the indicated drugs for the indicated hours. Thereafter, the cells were subjected to an XTT assay (Roche, catalog number: 11465015001) performed according to the manufacturer’s instructions. The absorbance of the formed XTT–formazan complex was quantitatively measured at 492 nm using an enzyme-linked immunoassay reader (Bio-Rad Laboratories, Inc.).

### Western Blot Analysis

The treated cellular protein extracts were prepared as described previously (Lin et al., 2017; Lee et al., 2018). In brief, equal amounts of protein were separated on an 8–10% SDS-PAGE gel and transferred onto polyvinylidene difluoride membranes. The membranes were blocked with 5% nonfat dried milk for 30 min and then incubated with primary antibodies for 6–12 h at room temperature. The following primary antibodies were used: anti-ACE2 (1:1,000; Cell Signaling), anti-STAT3 (1:1,000; Cell Signaling), anti-TMPRSS2 (1:1,000; Abcam), anti-β-actin (1:10,000; Santa Cruz), and anti-GAPDH (1:10,000; Santa Cruz) antibodies. All the primary and secondary antibodies were diluted with 1% nonfat dried milk in Tris-buffered saline with 0.1% Tween 20 detergent. The membranes were washed using 0.1% Tris-buffered saline with Tween-20 and incubated in horseradish peroxidase–conjugated secondary anti-mouse or anti-rabbit antibodies (Santa Cruz, ratio: 1:5,000) for 1 h at room temperature. The membranes were washed for 1 h at room temperature. Chemiluminescent protein signals were detected by applying the SuperSignal West Pico PLUS chemiluminescent substrate (Pierce, catalog number: 34087).

### Quantitative Real-Time Polymerase Chain Reaction

Quantitative real-time polymerase chain reaction (PCR) was performed as described previously (Lin et al., 2017; Lee et al., 2018). Total RNA of the indicated cells was extracted using the illustra™ RNAspin Mini RNA Isolation Kit (GE Healthcare, catalog number: 25-0500) and then reverse transcribed using the Superscript first-strand synthesis kit (Invitrogen, catalog number: 11904018)—all according to the manufacturer’s instructions. Quantitative real-time PCR analysis was performed using the comparative cycle threshold method on an ABI PRISM 7700 Sequence Detection System by using the SYBR Green PCR Master Mix kit, according to the manufacturer’s instructions. After initial incubation at 5°C for 2 min and then at 95°C for 10 min, 40 amplification cycles were performed at 95°C for 20 s, followed by 65°C for 20 s, and then 72°C for 30 s. *GAPDH* was used as the housekeeping gene for data normalization. Primers used were *ACE2* forward, 5'- TCC ATT GGT CTT CTG TCA CCCG-3' and *ACE2* reverse, 5'-AGA CCA TCC ACC TCC ACT TCTC-3', and *GAPDH* forward, 5'-TGC ACC ACC AAC TGC TTAGC-3' and *GAPDH* reverse, 5'-GGC ATG GAC TGT GGT CATGA-3'.

### Protein Quantification

For protein quantification, Western blot band images were analyzed using AlphaEase®FC according to the manufacturer’s instructions. After a band was selected for each group, the background was subtracted and the band densities were calibrated automatically. The density of the untreated group was used as the standard to calculate protein ratios for the other groups.

### Mice

All current procedures involving mice were approved by the Institutional Animal Care and Use Committee of Chang Gung Memorial Hospital (Approval number 2017081601). Surgery was performed under sodium pentobarbital anesthesia.

Ten 5- to 7-week-old male C57BL/6 mice (weight = 18–20 g), obtained from BioLASCO Taiwan, were randomized into two groups of five: one group received the vehicle (water), whereas the other received oral GB-1 at 200 mg/kg/day. Mouse weights were measured every 1–2 days for 1 week. At 1 week after treatment, the mice were sacrificed. Subsequently, mouse blood samples were tested for serum creatinine, aspartate aminotransferase, and alanine aminotransferase levels.

### Immunohistochemistry Assessment

Immunohistochemical (IHC) analysis was performed as described previously (Liu et al., 2019). Lung and kidney tissue specimens obtained from our mice were fixed with 4% formalin, embedded in paraffin, sectioned, and stained with primary antibodies against ACE2 (1:100; Bioss Antibodies) or STAT3 (1:100; Cell Signaling) or TMPRSS2 (1:1,000; Abcam). For IHC assessment, we used peroxidase-linked goat anti-rabbit secondary antibodies and the Rabbit Probe HRP Labeling Kit (catalog number: TAHC03D; BioTnA). The photomicrographs were observed under the Nikon TE3000 microscope. The ImageJ system (1.50 days, United States) was used to quantify the integrated optical density per stained area (IOD/area) for the staining of ACE2, STAT3, and TMPRSS2.

### High-Performance Liquid Chromatography

High-performance liquid chromatography (HPLC) analysis was performed on the LC-10Avp system (Shimadzu) equipped with a Supelco Discovery^®^ C18 column (5-μm particle size, 150-mm length × 4.6-mm internal diameter; Supelco); 0.1% phosphoric acid was used as the mobile phase; the flow rate was 0.6 ml/min; the detection wavelength was 280 nm; and the column temperature was 25°C.

### Statistical Analyses

All values are presented as means ± standard errors of the means of the replicate samples (*n* = 3–6, depending on the experiment). All experiments were repeated at least three times. Differences between the two groups were assessed using the unpaired two-tailed Student’s *t* test, and among those, more than two groups were examined using analysis of variance (ANOVA). For testing the significance of pairwise group comparisons, Tukey’s test was used as a post hoc test in ANOVA. For all comparisons, *p* values of <0.05 were considered to indicate statistical significance. SPSS (version 13.0; SPSS, Chicago, IL, United States) was used for all statistical analyses.

## Results

### Effect of GB-1 on HepG2, 293T, and Caco-2 Cell Growth and ACE2 and TMPRSS2 Expression

**FIGURE 1:**
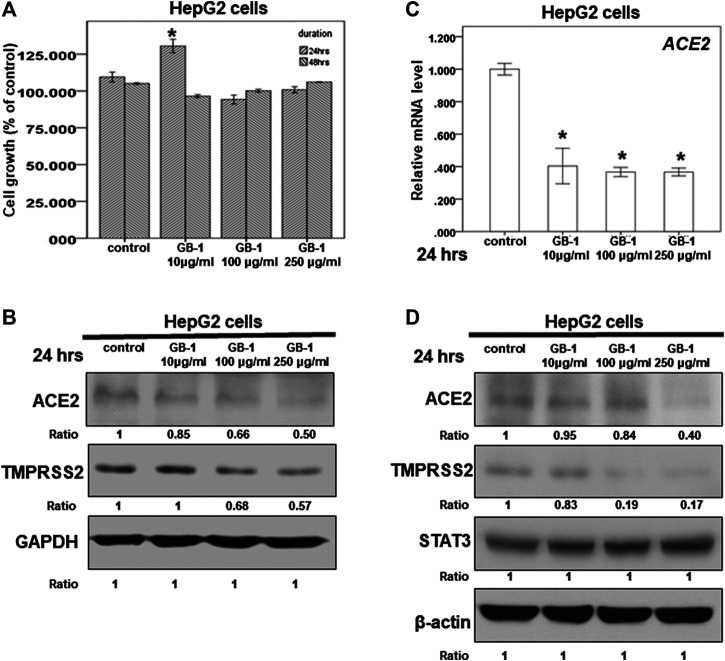
Effect of GB-1 on ACE2 and TMPRSS2 expression in HepG2 cells. **(A)** HepG2 cells were measured by XTT assay after indicated hours of culturing in the presence of GB-1. **(B,D)** Total cell extracts of HepG2 cells were harvested from untreated cells and cells treated with GB-1 for 24 h. The protein was immunoblotted with polyclonal antibodies specific for ACE2 or TMPRSS2 or STAT3. GAPDH or β-actin was used as an internal loading control. **(C)** Total mRNA was extracted from the HepG2 cells after treating with GB-1 for 24 h. The coding regions of human ACE2 were used as probes for real-time polymerase chain reaction analysis. All the results are representative of at least three independent experiments. (Error bars = mean ± S.E.M. * denotes samples significantly different from the control group with *p* < 0.05).

**FIGURE 2:**
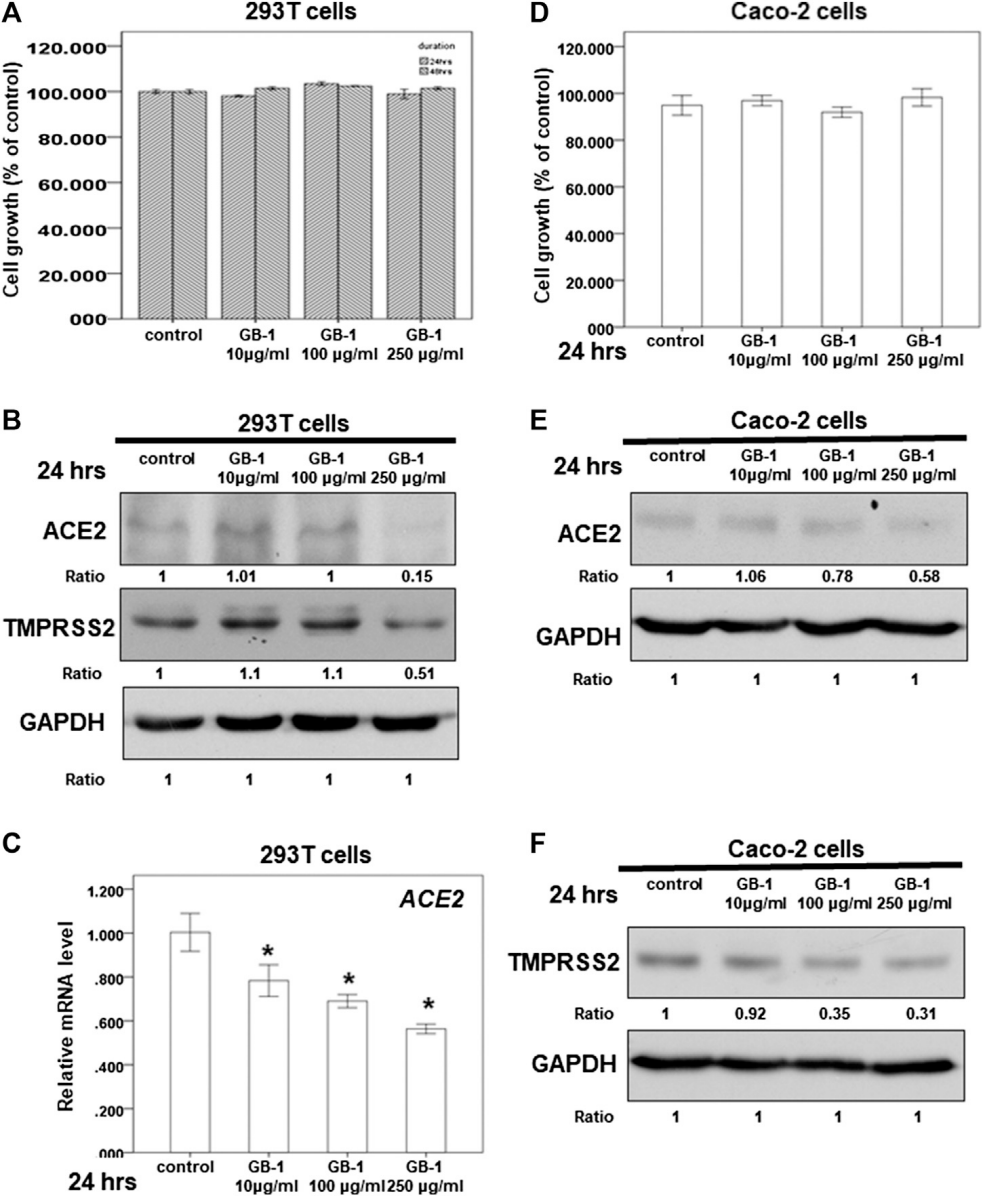
Effect of GB-1 on ACE2 and TMPRSS2 expression in 293T cells and Caco-2 cells. **(A**,**D)** 293T cells **(A)** or Caco-2 cells **(D)** were measured by XTT assay after indicated hours of culturing in the presence of GB-1. **(B**,**E,F)** Total cell extracts of 293T cells **(B)** or Caco-2 cells **(E,F)** were harvested from untreated cells and cells treated with GB-1 for 24 h. The protein was immunoblotted with polyclonal antibodies specific for ACE2 or TMPRSS2. GAPDH was used as an internal loading control. **(C)** Total mRNA was extracted from the 293T cells after treating with GB-1 for 24 h. The coding regions of human ACE2 were used as probes for real-time polymerase chain reaction analysis. All the results are representative of at least three independent experiments. (Error bars = mean ± S.E.M. * denotes samples significantly different from the control group with *p* < 0.05).

In previous studies, HepG2 cells and Caco-2 cells showed high ACE2 protein expression and were used as a model of SARS-CoV and SARS-CoV-2 entry models (Inoue et al., 2007; Liao et al., 2013; Bojkova et al., 2020). In addition, 293T cells have also been used as a model of SARS-CoV-2 entry (Letko et al., 2020). To investigate the effect of GB-1 on ACE2 and TMPRSS2 expression, we used HepG2 cells, 293T cells, and Caco-2 cells as cellular models. First, using an XTT assay, we investigated a potential cytotoxic effect of GB-1 on HepG2 cells, 293T cells, and Caco-2 cells. After treatment at a concentration range of 10–250 μg/ml, GB-1 had not inhibited the proliferation of HepG2 cells (Figure 1A) or 293T cells (Figure 2A) or Caco-2 cells (Figure 2D), suggesting that this concentration range of GB-1 has no considerable cytotoxic effect on HepG2 cell, 293T cell, and Caco-2 cell growth.

Because the SARS-CoV-2 spike protein can bind directly to ACE2 and is primed by TMPRSS2 (Hoffmann et al., 2020b; Walls et al., 2020; Yan et al., 2020), we examined the effect of GB-1 on ACE2 and TMPRSS2 expression in host cells. Twenty-four hours of treatment with GB-1 significantly reduced ACE2 and TMPRSS2 protein expression in HepG2 cells in a dose-dependent manner (Figures 1B,D). GB-1 also inhibited the protein expression of ACE2 and TMPRSS2 in 293T cells at a concentration of 250 μg/ml (Figure 2B). In addition, GB-1 inhibited the protein expression of ACE2 and TMPRSS2 in Caco-2 cells at a concentration of 250 μg/ml (Figures 2E,F). GB-1 also inhibited *ACE2* mRNA expression in HepG2 cells and 293T cells after 24 h (Figures 1C, 2C). In a recent study, silencing STAT3 (signal transducer and activator of transcription 3) can affect ACE2 expression (Shamir et al., 2020). In addition, inhibition of STAT3 with galiellalactone significantly reduced the expression of TMPRSS2 in benign tissue cultures (Handle et al., 2018). For investigating the mechanism, we examined the effect of GB-1 on STAT3 expression in host cells. The treatment with GB-1 had not affected STAT3 protein expression in HepG2 cells (Figure 1D). Overall, these results suggest that GB-1 can affect ACE2 and TMPRSS2 protein expression in HepG2 cells, 293T cells, and Caco-2 cells, without a significant cytotoxic effect.

### *In Vivo* Effect of GB-1 on the Mouse Model

**FIGURE 3:**
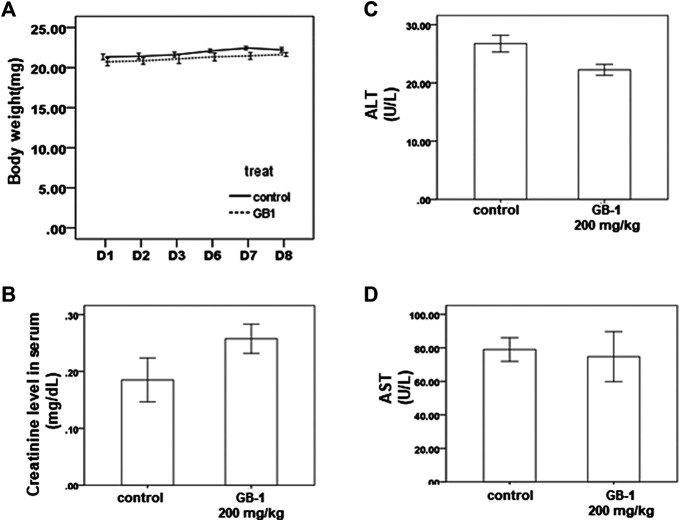
*In vivo* effect of GB-1 on the mouse model. **(A)** Average mice weights with vehicle/200 mg/kg/day GB-1 every day by oral administration over a time course of 1 week. **(B–D)** Creatinine **(B)**, ALT **(C)**, and AST **(D)** levels in serum of mice after the treatment of vehicle/GB-1. (*n* = 5 per group, error bars = mean ± S.E.M. * denotes samples significantly different from the control group with *p* < 0.05).

To investigate the effects of GB-1 *in vivo*, we used mice as our model. From the XTT assay, we revealed that 10–250 μg/ml of GB-1 had no significant cytotoxic effect on HepG2 cell, 293T cell, and Caco-2 cell growth (Figures 1A, 2A,D). We treated the mice with 200 mg/kg of GB-1 through oral administration every day. After 1 week of treatment, no significant alteration in either the activity or the body weight of the mice had been observed, and no mice had died (Figure 3A). Moreover, creatinine, alanine aminotransferase, and aspartate aminotransferase levels of serum in the GB-1 group were not elevated compared with those in the control group (Figures 3B–D), indicating that GB-1 did not cause significantly acute nephrotoxicity and hepatotoxicity in the mice.

**FIGURE 4:**
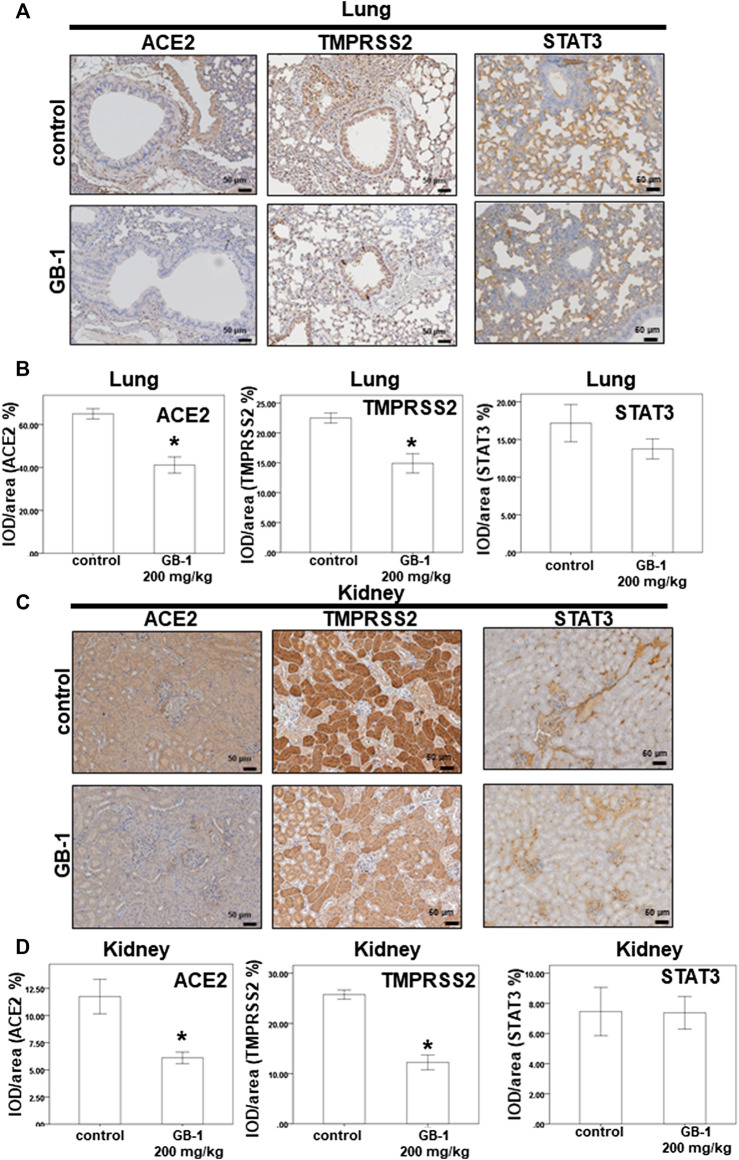
Effect of GB-1 on ACE2 and TMPRSS2 and STAT3 expression on the mouse model. **(A**,**C)** Representative IHC staining photomicrographs of the lung tissue **(A)** and kidney tissue **(C)** in mice. **(B**, **D)** Quantitative results of IHC staining, which were presented as the IOD/area and were proportional to the levels of ACE2 and TMPRSS2 and STAT3. (*n* = 5 per group, error bars = mean ± S.E.M. * denotes samples significantly different from the control group with *p* < 0.05).

As the lungs and kidneys have higher ACE2 expression and are the major target organs for SARS-CoV and SARS-CoV-2 infection (Hamming et al., 2004; Baig et al., 2020; Lukassen et al., 2020; Zou et al., 2020), we investigated the effect of GB-1 on ACE2 protein expression in the lung and kidney tissue. After 1 week of GB-1 treatment (200 mg/kg, oral administration), IHC data of the lung and kidney tissue showed that the ACE2 expression level in the GB-1 group was markedly reduced compared with that in the control group (Figure 4). In addition, the TMPRSS2 expression level in the lung and kidney tissue decreased substantially in the GB-1 group compared with the control group (Figure 4). However, the STAT3 expression level in the lung and kidney tissue showed no significant alteration between the GB-1 group and the control group (Figure 4). These results suggest that GB-1 may protect against SARS-CoV and SARS-CoV-2 infection through inhibiting ACE2 and TMPRSS2 expression in both lung and kidney tissues, without inducing significant nephrotoxicity or hepatotoxicity.

### Effect of *Glycyrrhiza uralensis* Fisch. ex DC. on ACE2 Expression

**FIGURE 5:**
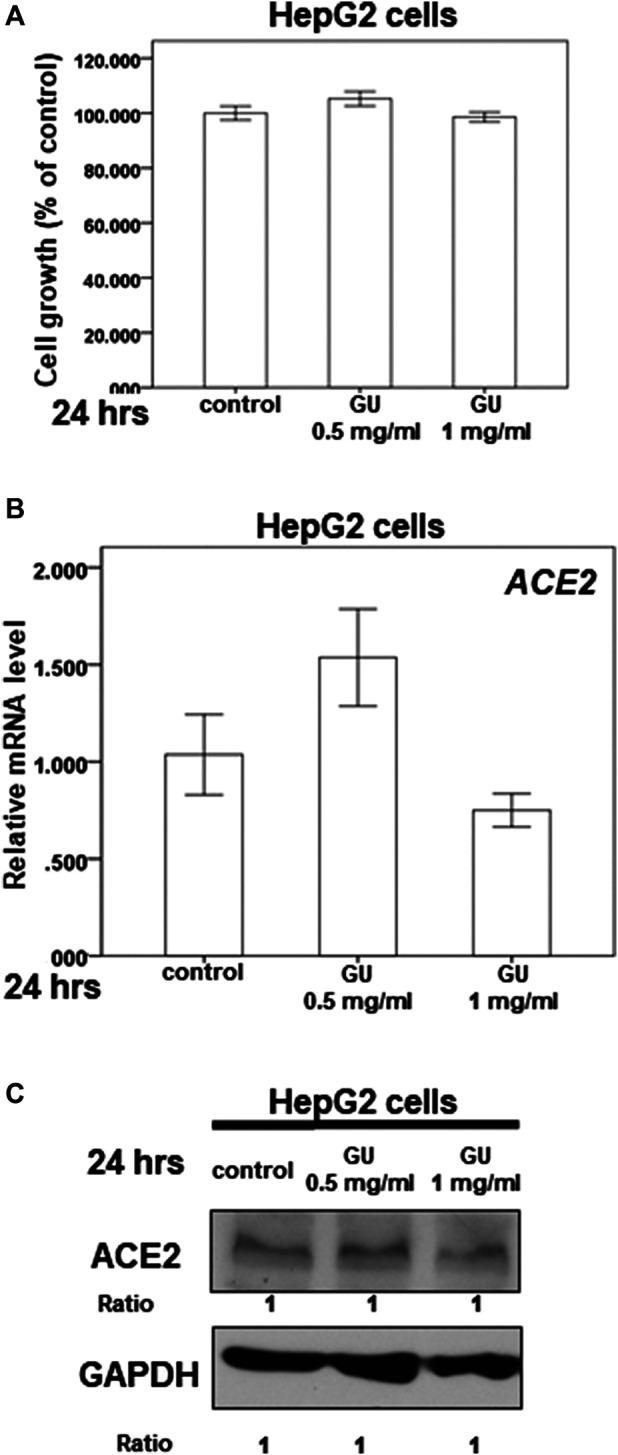
Effect of *Glycyrrhiza uralensis* Fisch. ex DC. extract (GU) on ACE2 expression. **(A)** HepG2 cells were measured by XTT assay after indicated hours of culturing in the presence of GU. **(B)** Total mRNA was extracted from the HepG2 cells after treating with GU for 24 h. The coding regions of human *ACE2* were used as probes for real-time polymerase chain reaction analysis. **(C)** Total cell extracts of HepG2 cells were harvested from untreated cells and cells treated with GU for 24 h. The protein was immunoblotted with polyclonal antibodies specific for ACE2 or TMPRSS2. GAPDH was used as an internal loading control. All the results are representative of at least three independent experiments. (Error bars = mean ± S.E.M. * denotes samples significantly different from the control group with *p* < 0.05).

The dry root of *Glycyrrhiza uralensis* Fisch. ex DC. (GU) and *Camellia sinensis* var. assamica extract are the major components of GB-1 and are commonly consumed in Taiwan and around the world for food and medicine. Our results indicate that 0.5–1 mg/ml of GU had no significant cytotoxic effect on HepG2 cell growth (Figure 5A). However, the same concentration range of GU could not inhibit *ACE2* mRNA and protein expression in HepG2 cells (Figures 5B,C). These results suggest that GU may not be responsible for the inhibitory effect of GB-1 on *ACE2* mRNA and protein expression in HepG2 cells.

### Effect of (+)-Catechin on ACE2 and TMPRSS2 Expression

**FIGURE 6:**
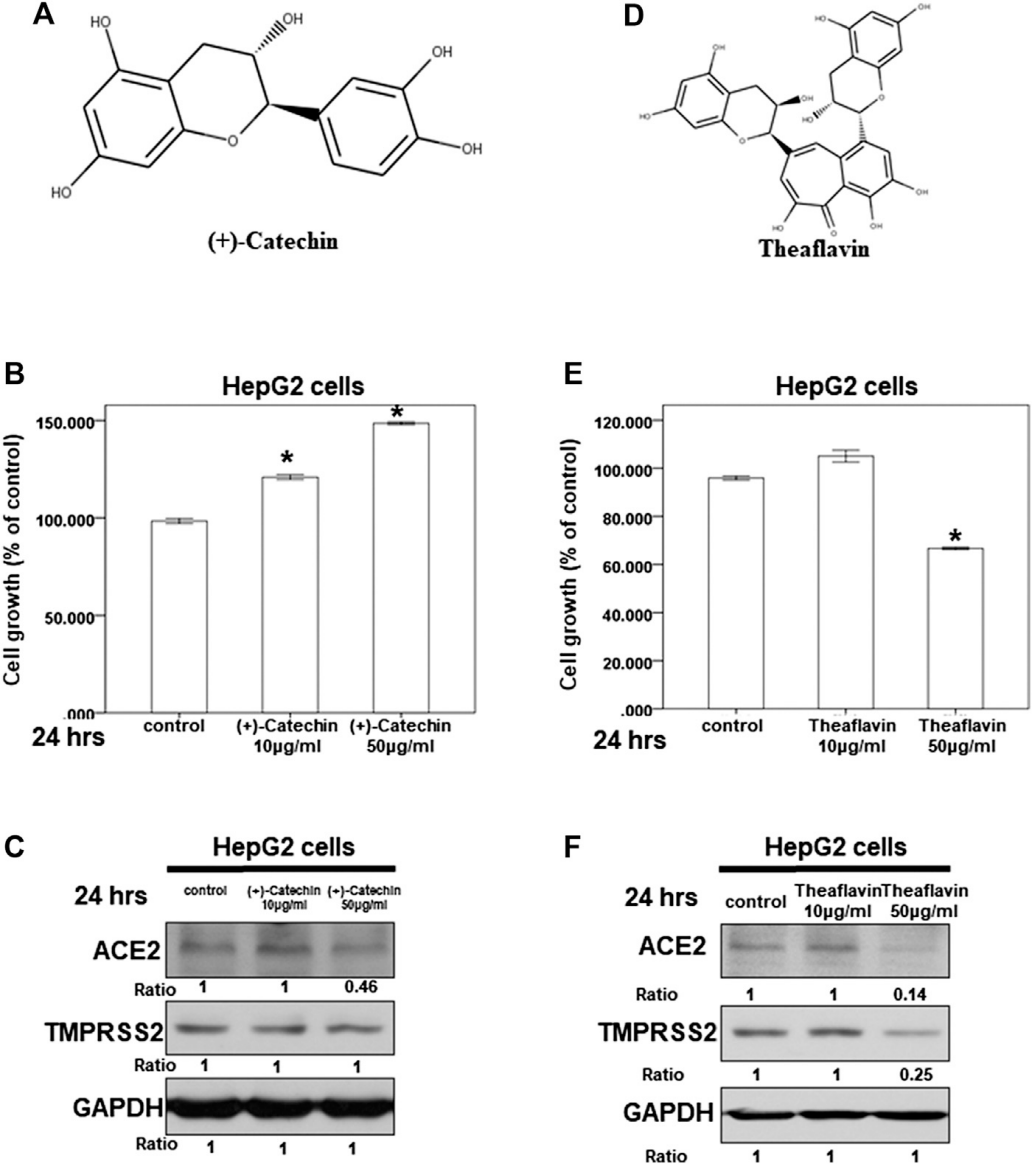
Effect of (+)-catechin and theaflavin on ACE2 and TMPRSS2 expression. **(A**,**D)** The structure of (+)-catechin **(A)** and theaflavin **(D)**. **(B**,**E)** HepG2 cells were measured by XTT assay after indicated hours of culturing in the presence of (+)-catechin **(B)** or theaflavin **(E) (C**,**F)** Total cell extracts of HepG2 cells were harvested from untreated cells and cells treated with (+)-catechin **(C)** or theaflavin **(F)** for 24 h. The protein was immunoblotted with polyclonal antibodies specific for ACE2 or TMPRSS2. GAPDH was used as an internal loading control. All the results are representative of at least three independent experiments. (Error bars = mean ± S.E.M. * denotes samples significantly different from the control group with *p* < 0.05).

Because we suspected that *Camellia sinensis* var. assamica extract might play a role in inhibiting the expression of ACE2 and TMPRSS2, we investigated the effect of (+)-catechin (Figure 6A), an index compound of *Camellia sinensis* var. assamica extract, on HepG2 cells. We first assessed the potential cytotoxicity of (+)-catechin in HepG2 cells at a concentration range of 10–50 μg/ml. Our results showed that (+)-catechin could not inhibit the growth of HepG2 cells (Figure 6B). However, 50 μg/ml of (+)-catechin inhibited ACE2 but not TMPRSS2 protein expression in HepG2 cells (Figure 6C). These results suggest that higher concentrations (≥50 μg/ml) of (+)-catechin can inhibit ACE2 protein expression in HepG2 cells.

### Effect of Theaflavin on ACE2 and TMPRSS2 Expression

Next, we investigated the effect of theaflavin (Figure 6D), another index compound of *Camellia sinensis* var. assamica extract, on HepG2 cells. We tested the potential cytotoxicity of theaflavin in HepG2 cells at a concentration range of 10–50 μg/ml. At 50 μg/ml, theaflavin had a mild cytotoxic effect on the growth of HepG2 cells (Figure 6E). Moreover, only 50 μg/ml of theaflavin inhibited ACE2 and TMPRSS2 protein expression in HepG2 cells (Figure 6F). These results suggest that higher concentrations (≥50 μg/ml) of theaflavin can inhibit the protein expression of ACE2 and TMPRSS2 in HepG2 cells.

### Effect of Theaflavin-3-Gallate on ACE2 and TMPRSS2 Expression

**FIGURE 7:**
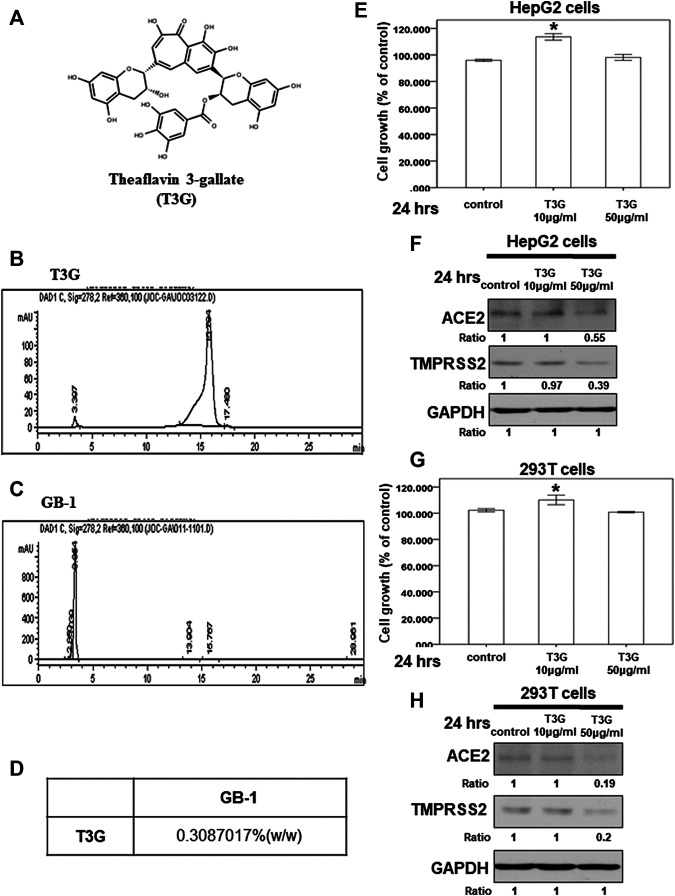
Effect of theaflavin-3-gallate on ACE2 and TMPRSS2 expression. **(A)** The structure of theaflavin-3-gallate. **(B**,**C)** HPLC chromatograms of theaflavin-3-gallate **(B)** and GB-.1 **(C) (D)** The concentration of theaflavin-3-gallate in GB-1. **(E**,**G)** HepG2 cells **(E)** and 293T cells **(G)** were measured by XTT assay after indicated hours of culturing in the presence of theaflavin-3-gallate. **(F**,**H)** Total cell extracts of HepG2 cells HepG2 cells **(F)** and 293T cells **(H)** were harvested from untreated cells and cells treated with theaflavin-3-gallate for 24 h. The protein was immunoblotted with polyclonal antibodies specific for ACE2 or TMPRSS2. GAPDH was used as an internal loading control. All the results are representative of at least three independent experiments. (Error bars = mean ± S.E.M. * denotes samples significantly different from the control group with *p* < 0.05).

We also investigated the effect of theaflavin-3-gallate (Figure 7A), another index compound of *Camellia sinensis* var. assamica extract, on HepG2 cells. In our HPLC result, the concentration of theaflavin-3-gallate in GB-1 is 0.3087017% (w/w) (Figures 7B–D). We tested the cytotoxicity of theaflavin-3-gallate in HepG2 cells and 293T cells and found that the theaflavin-3-gallate was not cytotoxic to the growth of HepG2 cells and 293T cells at a concentration of 10–50 μg/ml (Figures 7E,G). However, 50 μg/ml of theaflavin-3-gallate inhibited the protein expression of ACE2 and TMPRSS2 in HepG2 cells and 293T cells (Figures 7F,H). These results suggest that 50 μg/ml of theaflavin-3-gallate contributes to the inhibition of the protein expression of ACE2 and TMPRSS2.

## Discussion

The present study demonstrates that TMPRSS2 expression in host cells can activate SARS-CoV-2 and promote its spread (Hoffmann et al., 2020b). Camostat mesylate, an inhibitor of TMPRSS2 approved for clinical use, can block the SARS-CoV-2 infection of lung cells (Hoffmann et al., 2020b). However, studies involving mouse models of acute respiratory distress syndrome have reported that ACE2 knockout resulted in more severe symptoms (Imai et al., 2005). Higher levels of ACE2 in lung cells are associated with less severe acute respiratory distress syndrome (Wosten-van Asperen et al., 2013). The disruption of the physiological balance between ACE/ACE2 and the angiotensin II/angiotensin system by SARS-CoV infection plays a pathogenic role in SARS-CoV–induced lung injury (Kuba et al., 2006; Yamamoto et al., 2006; Imai et al., 2008; Wu, 2020). Therefore, developing simultaneous inhibitors of both ACE2 and TMPRSS2, rather than only ACE2 inhibitors, may be a more suitable strategy for blocking SARS-CoV-2 infection. Our finding that GB-1 and theaflavin-3-gallate can inhibit the protein expression of both ACE2 and TMPRSS2 indicates that they could be candidates for the prophylaxis or treatment of SARS-CoV-2 infection in the further. In previous studies, STAT3 can affect both the expression of ACE2 and TMPRSS2 (Handle et al., 2018; Shamir et al., 2020). However, our studies showed GB-1 had not affected STAT3 protein expression in HepG2 cells and lung and kidney tissues of mice (Figures 1D, 4). These results suggested GB-1 might affect the expression of ACE2 and TMPRSS2 through other signal pathways.

In our previous study, we discovered that theaflavin has a potential chemical structure of anti-SARS-CoV-2 RNA-dependent RNA polymerase (Lung et al., 2020). In addition, 50 μg/ml of theaflavin was responsible for inhibiting the protein expression of ACE2 and TMPRSS2 in HepG2 cells. Chen et al. (2005) reported that theaflavin-3-gallate inhibited the 3C-like protease activity of SARS-CoV. In another study, (−)-catechin gallate and (−)-gallocatechin gallate demonstrated remarkable inhibition of SARS-CoV nucleocapsid protein (Roh, 2012). Nguyen et al. (2012) also reported that epigallocatechin gallate and gallocatechin gallate, which belong to the catechin family, had good inhibition of the 3C-like protease of SARS-CoV. In our study, 50 μg/ml of theaflavin-3-gallate, theaflavin, and (+)-catechin inhibited the protein expression of ACE2, and 50 μg/ml of theaflavin-3-gallate and theaflavin inhibited TMPRSS2 protein expression. Glycyrrhizin, the bioactive compound in GU, inhibited SARS-CoV replication (Cinatl et al., 2003; Hoever et al., 2005). Our study discovered GU extract was not responsible for inhibiting *ACE2* mRNA and protein expression in HepG2 cells in the concentration range of 0.5–1 mg/ml. However, many compounds in GB-1, including GU and *Camellia sinensis* var. assamica, remain unstudied. Some of these compounds might play important roles in the effect of ACE2 and TMPRSS2. These compounds may work together to inhibit the protein expression of ACE2 and TMPRSS2.

In conculsion, GB-1 is a potential candidate for prophylaxis of SARS-CoV-2 infection through inhibiting protein expression of ACE2 and TMPRSS2. Some index compounds of *Camellia sinensis* var. assamica, including theaflavin-3-gallate, theaflavin, and (+)-catechin, may be essential to inhibiting the protein expression of ACE2 and TMPRSS2. However, the exact clinical effect remains unclear; further studies are necessary to confirm the protective effects of GB-1 and theaflavin-3-gallate against SARS-CoV-2 entry.

## Data Availability Statement

The raw data supporting the conclusions of this article will be made available by the authors, without undue reservation, to any qualified researcher.

## Ethics Statement

The animal study was reviewed and approved by the Institutional Animal Care and Use Committee of Chang Gung Memorial Hospital (Approval number 2017081601).

## Author Contributions

C-YW conceived the idea and designed experiments and wrote manuscript. Y-SL prepared GB-1 and performed the experiments; L-HS, Y-CC, and H-TL analyzed the data. Y-HY revised English writing of the manuscript. All authors reviewed and approved the final version.

## Funding

This work was supported by MOST 108-2320-B-182-021 from Ministry of Science and Technology and CORPG6K0211 from Chang Gung Memorial Hospital (Chiayi) to C-YW.

## Conflict of Interest

The authors declare that the research was conducted in the absence of any commercial or financial relationships that could be construed as a potential conflict of interest.

## Abbreviations

ACE2, angiotensin-converting enzyme 2; TMPRSS2, transmembrane protease, serine 2; STAT3, signal transducer and activator of transcription 3; SARS-CoV-2, severe acute respiratory syndrome coronavirus 2; SARS-CoV, severe acute respiratory syndrome coronavirus; COVID-19, coronavirus disease 2019; IHC, immunohistochemistry; GU, *Glycyrrhiza uralensis* Fisch. ex DC.; T3G, theaflavin-3-gallate.
